# Mitochondrial Neurogastrointestinal Encephalopathy: A Case Report from Northeast of Iran

**DOI:** 10.34172/mejdd.3386

**Published:** 2026-04-30

**Authors:** Masoud M Malekzadeh, Maryam Anvari, Hadi Bagherihosseini, Azita Ganji

**Affiliations:** ^1^Department of Gastroenterology and Hepatology, Faculty of Medicine, Mashhad University of Medical Sciences, Mashhad, Iran; ^2^Digestive Disease Research Center, Digestive Disease Institute, Shariati Hospital, Tehran, Iran

**Keywords:** Mitochondrial neurogastrointestinal encephalopathy, Gastrointestinal dysmotility, Iran

## Abstract

Mitochondrial neurogastrointestinal encephalopathy (MNGIE) is a rare autosomal recessive disorder often misdiagnosed due to its heterogeneous presentation. We describe a 21-year-old man with a long history of episodic nausea and vomiting, weight loss, and intermittent diarrhea since childhood, initially misdiagnosed as Crohn’s disease following a colonoscopy-induced perforation. Despite treatment with adalimumab, his gastrointestinal symptoms persisted. Subsequent investigations revealed dilated stomach and duodenum, and new neurological findings (ptosis, nasal speech) prompted further evaluation. Brain magnetic resonance imaging (MRI) showed leukoencephalopathy, leading to the definitive diagnosis of MNGIE. This case highlights the importance of considering mitochondrial disorders in young patients with refractory gastrointestinal dysmotility and concomitant neurological features to prevent diagnostic delays and unnecessary interventions.

## Introduction

 Mitochondrial neurogastrointestinal encephalopathy (MNGIE) is an ultra-rare multisystem disorder caused by mutations in the *TYMP* gene, leading to impaired nucleotide metabolism and characteristic clinical features: severe gastrointestinal dysmotility, cachexia, ptosis, ophthalmoparesis, peripheral neuropathy, and leukoencephalopathy.^1,2^ The initial presentation is often gastrointestinal, mimicking more common conditions like functional disorders. Diagnostic delays are frequent, leading to inappropriate treatments and procedures. We present this case to emphasize that neurological symptoms in conjunction with gastrointestinal symptoms should prompt clinicians to consider MNGIE in the differential diagnosis.

## Case Report

 A 21-year-old unemployed man presented with a long-standing history of episodic nausea and vomiting dating back to childhood, occurring every 1-2 months. The frequency of vomiting intensified 3 years prior to admission, accompanied by postprandial symptoms and significant weight loss. He also complained of intermittent diarrhea and bloating. During a diagnostic workup, he underwent a colonoscopy that resulted in colonic perforation, requiring emergency surgery. Postoperatively, he developed an enterocutaneous fistula. A provisional diagnosis of Crohn’s disease was made, and treatment with adalimumab (Cinnora®) was initiated with intermittent antibiotics. The fistula closed after 6 months. However, over the next 2 years, his vomiting and postprandial abdominal pain persisted, and he failed to gain weight. Due to the lack of improvement and negative crypt destruction in colonic tissue, he was referred for further evaluation.

 On examination, vital signs were stable. Physical examination revealed unilateral ptosis, a midline laparotomy scar and mild epigastric distention. Neurological examination confirmed ptosis and a notable nasal speech.

 The laboratory test was unremarkable. Fecal calprotectin was in the normal range. Immunodeficiency workup was negative (Table 1).

**Table 1:** Laboratory findings of the patient in the last referral to our center

**Lab**	**Result**
WBC (Neutrophil%)	5700 cell/mm^3^ (47)
Hb (MCV)	14 g/dL (92)
Plt	182000 cell/mm^3^
FBS	80 mg/dL
CRP	2 mg/dL
ESR	13 mm/hr
AST	37 IU/L
ALT	43 IU/L
ALP	183 IU/L
LDH	628 IU/L
Urea	26 mg/dL
Creatinine	0.9 mg/dL
Albumin	4.3 g/dL
Protein	7.1 g/dL
Sodium	142 mEq/L
Potassium	4.1 mEq/L
Magnesium	2.5 mg/dL
S/E	WBC = 0-1, RBC = 0-1
Calprotectin	120 ug/g
Serum protein electrophoresis	Normal
HIV Ab	Negative

WBC: White blood cell, Hb: Hemoglobin, MCV: Mean corpuscular volume, Plt: Platelet, FBS: Fasting Blood Sugar, CRP: C-reactive protein, ESR: Estimated sedimentation rate, AST: Aspartate transferase, ALT: Alanine transferase, ALP: Alkaline phosphatase, LDH: Lactate dehydrogenase, S/E: Stool exam

 Abdominal CT enterography revealed significant dilatation of the stomach, bulb, and second part of the duodenum, but showed no evidence of small bowel involvement suggestive of Crohn’s disease (Figure 1).

**Figure 1:**
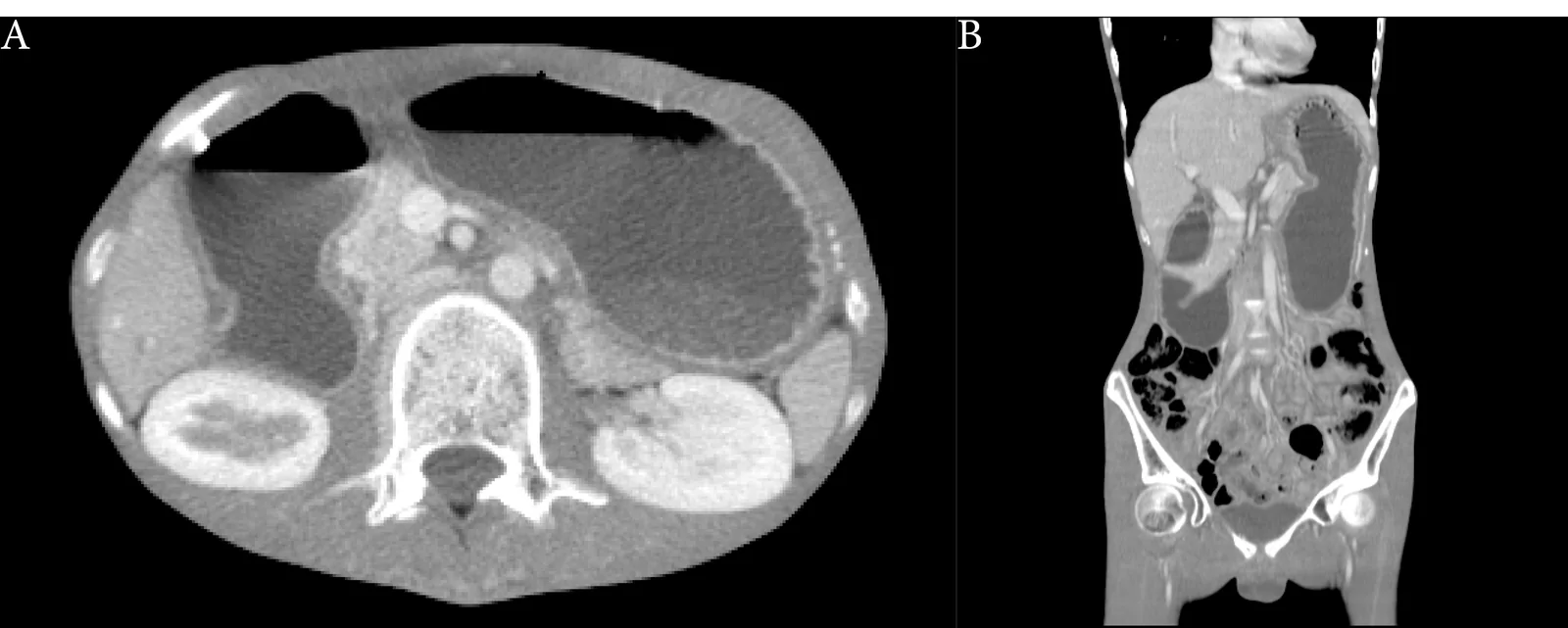
Abdominal CT scan axial view (A) and coronal view (B) revealed dilated stomach, bulb, and D2 with no evidence of an obvious luminal obstructive lesion

 The findings suggested possible adhesion bands affecting the third part of the duodenum and proximal dilation. Therefore, push enteroscopy was done, but no luminal obstruction was found. However, nodularity of the mucosa in the bulb, D2, and D3 was noted (Figure 2).

**Figure 2:**
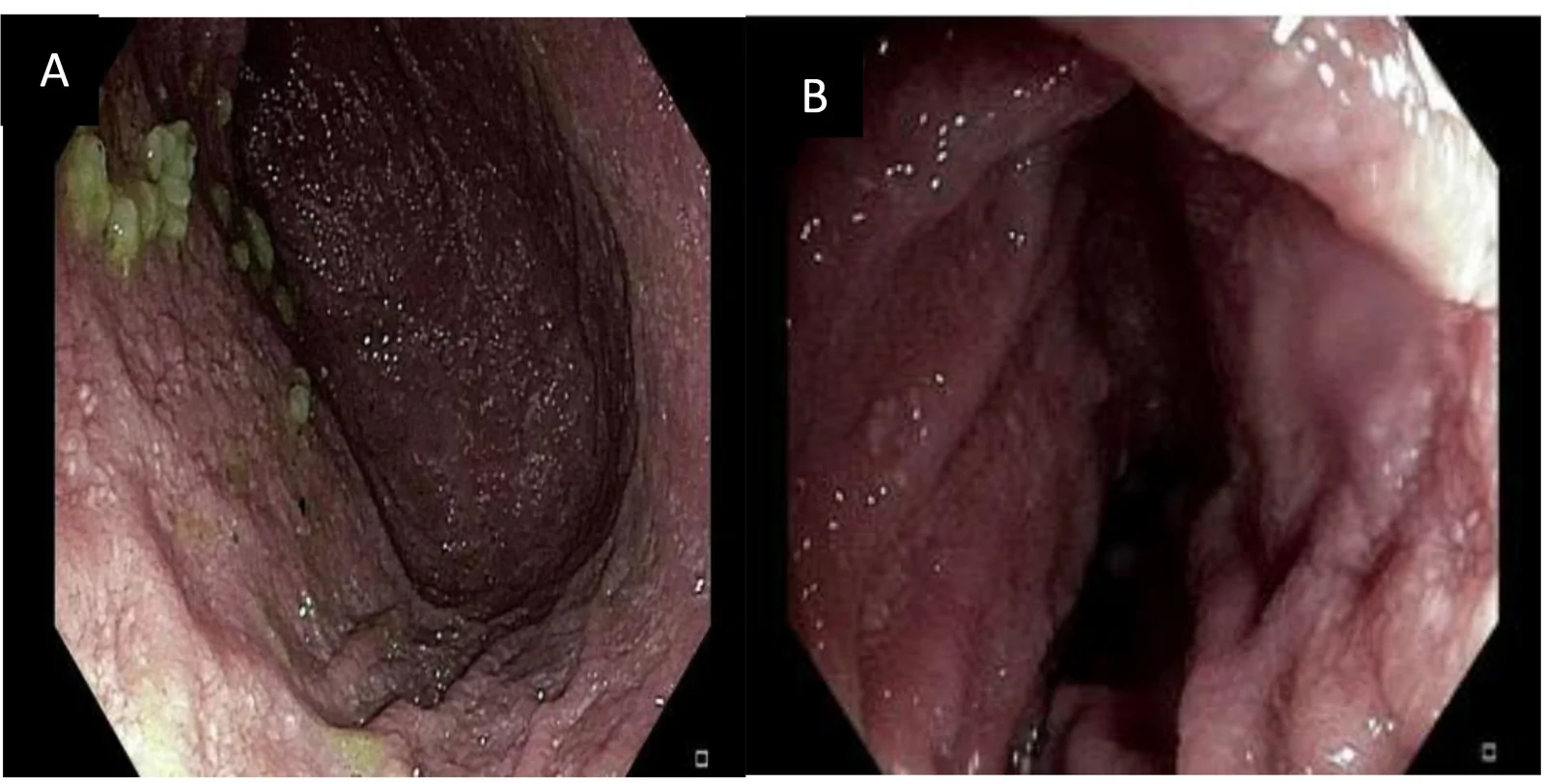
Push enteroscopy showed a dilated D2 with mucosal nodularity (A). The scope passed to D3 without pressure and showed mucosal nodularity on the third part of the duodenum (B)

 Multiple biopsies of the duodenal mucosa showed normal villous architecture with increased intraepithelial lymphocytes. There was no evidence of granulomas or ulceration in favor of Crohn’s disease.

 Due to a lack of notable luminal stricture and normal laboratory values in addition to ptosis, the patient was referred to the neurology clinic for further investigation. A brain MRI was done, which showed leukoencephalopathy (Figure 3).

**Figure 3:**
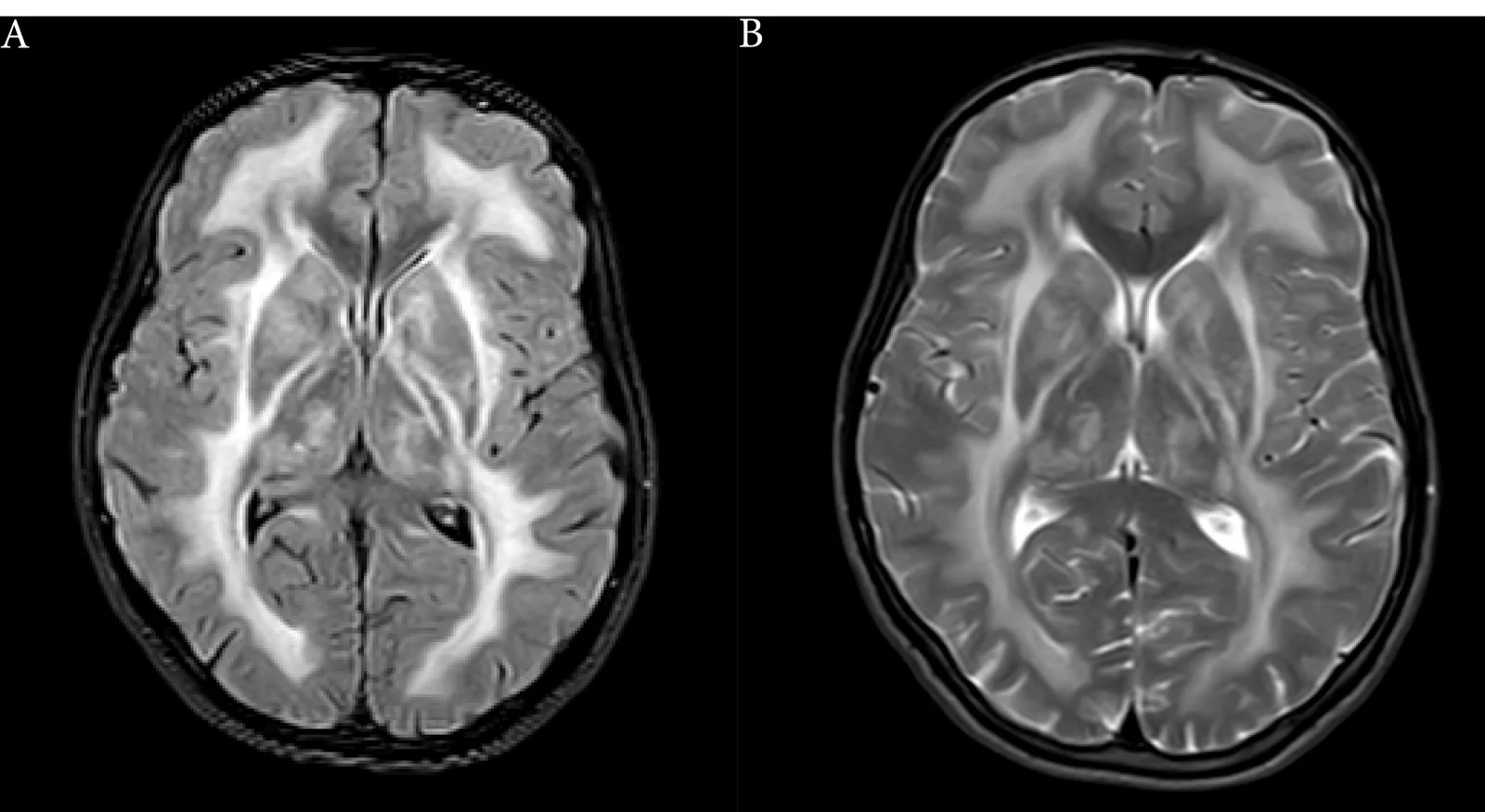
Brain MRI showing the evidence of symmetrical hyperintensity in white matter in both hemispheres was noted in the T2-weighted image (A). Also, there were areas of hyperintensity in both the basal ganglia and the thalamus in the T2-weighted image (B)

 The final diagnosis was MNGIE. This disease is diagnosed based on the classic triad of chronic intestinal pseudo-obstruction (refractory vomiting, gastric/duodenal dilatation), neurological manifestations (ptosis, nasal speech suggestive of peripheral neuropathy), and radiological evidence of leukoencephalopathy on MRI.

 The patient’s previous treatment with adalimumab was discontinued as it was not indicated. For symptomatic management, he was started on pantoprazole and metoclopramide. However, he showed no significant clinical response. The patient’s condition remained stable without improvement in 6 months of follow-up thereafter, reflecting the progressive and treatment-resistant nature of MNGIE. He was referred for specialized genetic counseling and evaluation for potential experimental therapies.

## Discussion

 We reported a young man with a long history of periodic nausea and vomiting, which had exacerbated in the recent 3 years with concomitant minor neurological findings. This case illustrates a classic yet often missed presentation of MNGIE. As seen in this case, surgical interventions in MNGIE are prone to complications due to baseline motility disorder and should be avoided unless absolutely necessary ^3^. The key to the correct diagnosis was the integration of persistent gastrointestinal dysmotility resistant to standard inflammatory bowel disease (IBD) therapy with the emergence of subtle neurological signs. The normal calprotectin and CT enterography effectively ruled out active Crohn’s disease, and the brain MRI definitely made the diagnosis.

 Age of disease presentation differs among patients with MNGIE, ranging from 5 months to 43 years, but similar to our case, mostly present at young adulthood ^2^. The average diagnostic delay is about 10 years ^1^. Gastrointestinal symptoms were the most common initial symptoms in previous case series ^4,5^. As in this case, who presented with prominent nausea and vomiting and gastroparesis, delayed gastric emptying and small intestinal dysmotility were the most common gastrointestinal symptoms ^2^. MRI has a fundamental role in the diagnosis of MNGIE; although not exclusive, signal intensities increase within cerebral white matter on T2-weighted images ^6^. There are different proposed treatment approaches for this disease, none of which has been fully successful, such as hematopoietic stem cell therapy, gene therapy, dialysis, and liver transplantation ^7^.

 Although not the first reported MNGIE case in Iran ^8-11^ it would be added to the previous reported cases. This case taught us to consider mitochondrial disorders in any young patient with chronic, refractory gastrointestinal pseudo-obstruction. Also suggesting that we actively seek and recognize concomitant neurological signs (ptosis, ophthalmoparesis, neuropathy, hearing loss) in patients with unexplained gastrointestinal symptoms.

## Conclusion

 MNGIE is a devastating disease that requires a high index of suspicion for diagnosis. In cases of treatment-refractory gastrointestinal dysmotility, especially when accompanied by neurological features, MNGIE must be included in the differential diagnosis. Early genetic confirmation can prevent unnecessary treatments, invasive procedures, and provide accurate genetic counseling, which may improve patient management.
